# Carbon emission and water use efficiency response to tillage methods and planting patterns of winter wheat in the North China Plain

**DOI:** 10.7717/peerj.9912

**Published:** 2020-09-15

**Authors:** Yuzhao Ma, Quanqi Li

**Affiliations:** College of Water Conservancy and Civil Engineering, Shandong Agricultural University, Tai’an, China

**Keywords:** Carbon emission, No tillage, Water use efficiency, Wide-precision planting, Winter wheat

## Abstract

**Background:**

Implementing sustainable farming practices for winter wheat (*Triticum aestivum* L.) in the North China Plain may be a way to reduce carbon emissions. No tillage generally results in less net CO_2_ loss from farmland, but no tillage also reduces the grain yield and water use efficiency (WUE) of winter wheat. Wide-precision planting of winter wheat may enhance the grain yield and WUE; however, it is not known precisely how tillage and planting patterns affect CO_2_ exchange, grain yield and WUE.

**Methods:**

In this study, two tillage methods (conventional tillage, T and no tillage, NT) and two planting patterns (conventional planting, C and wide-precision planting, W) were used in two consecutive winter wheat growing seasons.

**Results:**

Compared with the T treatments, the NT treatments had significantly lower cumulative net CO_2_ emissions in 2015–2016 and 2016–2017 (30.8 and 21.3%, respectively), and had lower grain yields (9.0 and 9.4%, respectively) and WUE (6.0 and 7.2%, respectively). The W treatments had a compensating effect on grain yield failure and reduced cumulative net CO_2_ emissions more than C treatments, thereby increasing WUE, reducing carbon emissions per unit water consumption, and increasing the yield carbon utilization efficiency (YCUE). The lowest cumulative CO_2_ emissions and highest YCUE were observed for NT with W treatment. Results from this analogous tillage experiment indicated that NT and W farming practices provide an option for reducing carbon emissions and enhancing WUE and YCUE for sustainable winter wheat development.

## Introduction

Winter wheat (*Triticum aestivum* L.), a vital grain crop, feeds more than 35% of the global population and is significant for nutritional security (Wakchaure et al., 2016). Water is the main limiting factor for winter wheat production because of uneven distribution of precipitation and overexploitation of groundwater resources. China is the largest producer of winter wheat (Daryanto, Wang & Jacinthe, 2016). The North China Plain (NCP), which accounts for about 25% of national food production, is one of the largest winter wheat planting regions. There is less than 200 mm of precipitation in the NCP during the winter wheat growing season, which cannot meet the water demands of winter wheat (300**–**400 mm) (Ren et al., 2018a; Ren et al., 2018b). Aridity is predicted to increase globally: around 20–30% of the total land surface will be classed as arid with the global temperatures rise 2 °C, and droughts will become more frequent (Park et al., 2018).

Understanding the potential effects of global warming on the environment, agriculture, resources, and energy utilization is a priority for research (Zhang et al., 2013). Greenhouse gas (GHG) emissions are the major contributor to global warming (IPCC, 2014). Soil forms the second largest carbon pool after the oceans, and emissions from soil are the second largest anthropogenic source of CO_2_ (Quere et al., 2009). Agricultural lands occupy approximately 40**–**50% of the total land surface, and agriculture contributes 22% of total emissions (IPCC, 2014). Agricultural GHG emissions are increasing by 0.06 Pg CO_2_-eq per year. GHGs emitted directly from the soil account for 76**–**85% of the total soil carbon emissions, mostly during the growing seasons (Cui et al., 2019). Studies of soil CO_2_ emissions from China’s winter wheat fields are therefore of national and global significance for estimating GHG emissions and greenhouse effects (Hou et al., 2019). The relationship between cleaner agricultural production and environmental influences are a factor for scientists and policy makers (Lal, 2003).

Sustainable land use and management practices are effective methods to limit the amount of GHG emissions from the soil carbon pool (Schimel et al., 2001). The choice of tillage method influences soil properties and soil organic carbon allocation, which in turn affects GHG emissions (Ussiri & Lal, 2009). Conservation tillage reduces soil erosion, saves water, reduces labor, and improves soil quality. The no tillage (NT) method is considered a viable way to decrease CO_2_ emissions. A previous study found that, compared to conventional tillage (T), NT reduced carbon emissions by 22% in Italian durum wheat production (Alhajj et al., 2017). However, in the NCP, while NT practices have been shown to reduce GHG emissions, winter wheat grain yield and water use efficiency (WUE) were much lower in NT treatments than in T treatments (Tian et al., 2013; Guan et al., 2015). In the NCP, fewer spike numbers were observed in NT and this reduced winter wheat grain yield (Ren et al., 2018a; Ren et al., 2018b). Understanding the responses of grain yield, GHG emissions, and WUE under different tillage methods is important to establish a sustainable wheat supply.

Optimizing planting strategies can help increase crop yield and WUE. Research found that adopting wide–precision planting (W) of 6**–**8 cm sowing width, instead of 3**–**5 cm, increased photosynthetically active radiation capture ratio at 40 and 60 cm above the ground, and it improved spike numbers, which increased winter wheat growth and grain yield (Zhao et al., 2013). In a large area of the NCP, the W approach for winter wheat produced higher grain yields (Yu et al., 2010). Under well-irrigated conditions, W increased winter wheat grain yield compared with that of conventional planting (C) (Zhao et al., 2013). Compared with conventional planting, wide-precision planting enhances grain yields because it changed yield components especially increasing spike numbers and improving WUE (Li et al., 2015). However, the effect of different planting patterns and tillage methods on GHG emissions remains unclear.

One limiting factor for GHG emissions is soil moisture content (Zhang et al., 2010). NT minimizes soil disturbance and promotes soil water conservation, which minimizes CO_2_ gas diffusion from the soil to the atmosphere (Tu et al., 2017). The coupling of evapotranspiration and carbon absorption is the essence of WUE, which reflects the relationship between water consumption and carbon sequestration (Keenan et al., 2013). Liu et al. (2014) found that planting patterns affected the carbon utilization of a crop, and Hu et al. (2017) used carbon emission per unit water consumption (WUE_CE_) to express the relationship between evapotranspiration and carbon emissions.

It is known that NT reduces GHG emissions but often leads to decreases in grain yield, whereas W has been used widely in the NCP to maximize winter wheat production and WUE. However, few studies have investigated the effects of W under NT with respect to GHG emissions and water use. We hypothesized that combining W with NT could mitigate the reductions in winter wheat grain yields and WUE under NT conditions while still reducing carbon emissions. In this study, we combined two tillage methods (NT and T), and two planting patterns (W and C) to (1) determine the effects of W under NT on CO_2_ emissions and grain yield; (2) identify the WUE, yield carbon utilization efficiency (YCUE), and WUE_CE_ under W and NT conditions; and (3) clarify the compensatory effect of W under NT on grain yield and WUE. These results could support the sustainable development of winter wheat and mitigate trends in global warming.

## Materials and Methods

### Study site and soil properties

**Figure 1:**
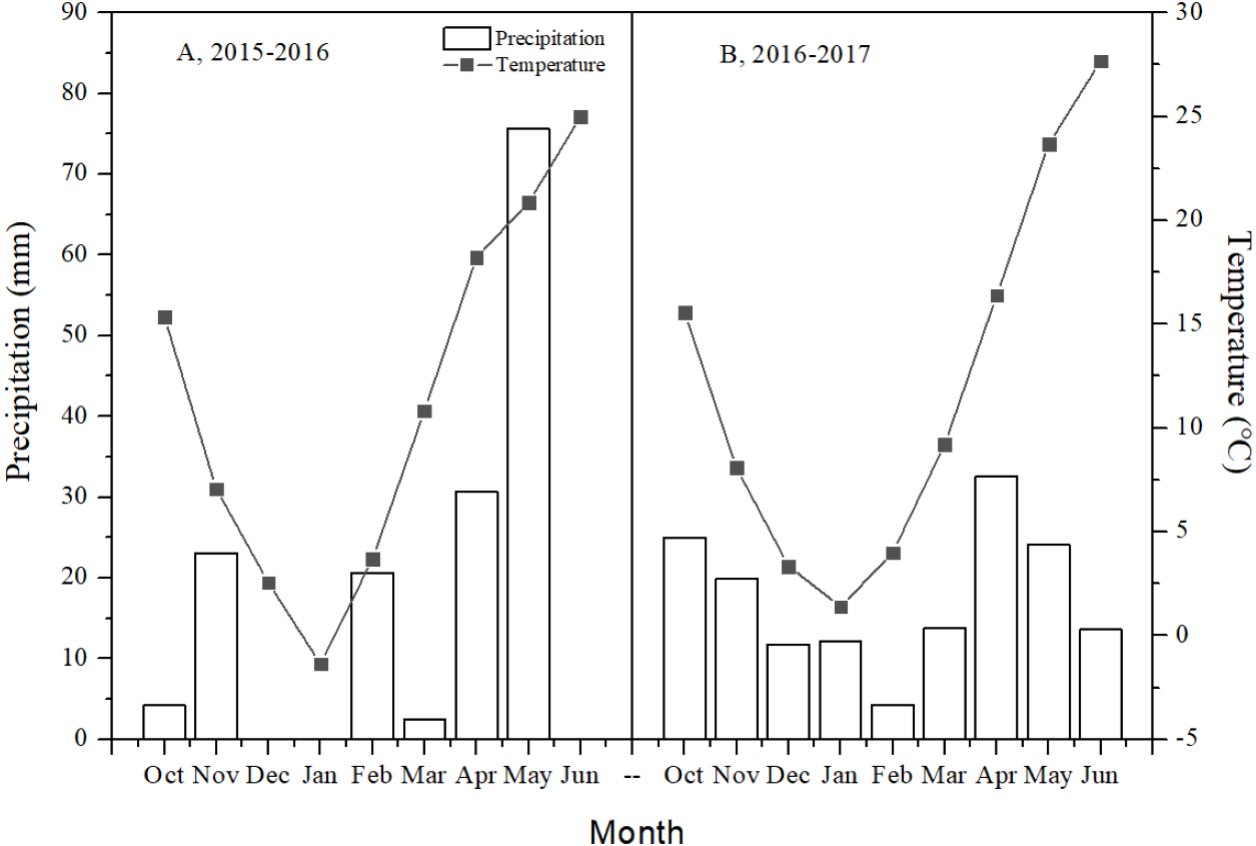
Monthly total precipitation and mean monthly air temperature in the 2015–2016 and 2016–2017 winter wheat growing seasons, at the Agricultural Experiment Station of Shandong Agricultural University. Total precipitation in October was from seeding time to end of October and total precipitation in June was from beginning of June to the harvest time.

The field experiment was conducted at the Experimental Station of Shandong Agricultural University (117°9′03″E, 36°10′9″N) in the NCP from 2015 to 2017. The study area is characterized as a temperate continental monsoon climate. During the two experimental years (2015–2016 and 2016–2017), total precipitation in the growing season was 156.7 and 157.3 mm, respectively (Fig. 1).

Winter wheat was planted in 3.0 m ×3.0 m study plots under field conditions. The soil was classified as loamy clay. The available potassium, available phosphorus, and the available nitrogen content were 92.6, 16.2, and 108.3 mg kg^−1^, respectively, in the topsoil layer (0**–**20 cm).

### Study designs and management

**Figure 2:**
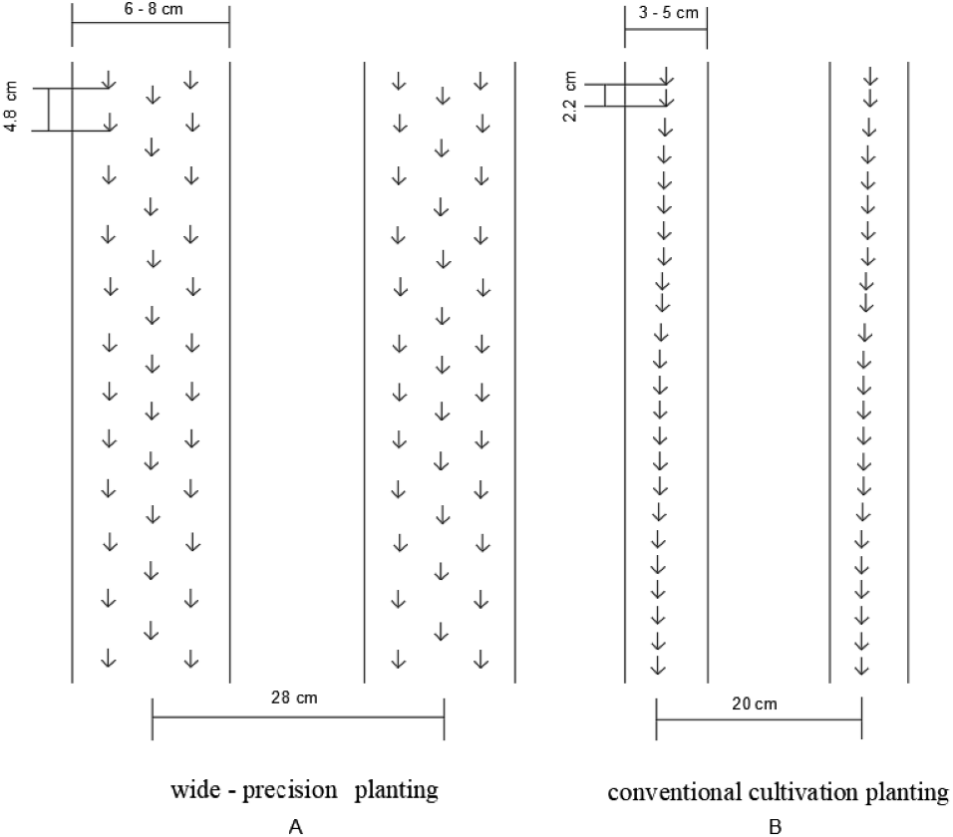
Schematic diagram of wide-precision planting (W) and conventional planting (C) in this study.

The two tillage methods (conventional tillage, T and no tillage, NT) and two planting patterns (conventional planting, C and wide-precision planting, W) were arranged in random block design with three replicates each, for a total of 12 plots. The experiment began in 2015. The T treatments were manually ploughed to a depth of 25 cm using a shovel on 7 Oct 2015 and 6 Oct 2016, respectively. Soil in the NT treatment are not ploughed. The previous crop was summer maize, and maize straw was removed before sowing in both tillage methods. The sowing and row spacing of W and C are shown in Fig. 2. The sowing space for the W treatments was dug with an 8 cm hoe, while the C treatments was dug with a 5 cm hoe.

   We used the Jimai 22 with high yield winter wheat cultivar. It was sown at a seeding rate of 222 grain m^−2^ by hand on 8 Oct 2015 and 7 Oct 2016. Each plot was given the same amount and type of fertilizers [potassium phosphate (30.0 g m^−2^), potassium chloride (7.5 g m^−2^) and urea (15.0 g m^−2^)] with irrigation (60 mm) before sowing. At the jointing stage (17 Mar 2016 and 15 Mar 2017), additional urea (15.0 g m^−2^) combined with irrigation (60 mm) was applied. The amount of irrigation was controlled using a flow meter. Winter wheat was harvested on 1 Jun 2016 and 3 Jun 2017.

### Cumulative CO_2_-C emissions

A gas analyzer (GHX–305; ADC Bio–scientific Ltd., Hoddesdon, UK) was used to measure the CO_2_ flux. It was composed of a PVC pipe (the pipe size was 25.0 cm diameter and 15.7 cm height), covered with a lid to form a dark chamber. The chamber was placed on the surface of the soil before measurement and pressed down to ensure a sealed environment. The seal was maintained to ensure air tightness during measurements. Measurements were taken from 9:00 to 10:00 AM on sunny days, at the seeding, wintering, jointing, heading, milking, maturity stage per experimental year. We allowed a 2 min wait for data stabilization before each reading.

Cumulative CO_2_-C emissions were computed following the method (Liu et al., 2014),

(1) \begin{eqnarray*}\mathrm{E}= \frac{V}{A} \times 100\times \rho \times \frac{\mathrm{dc}}{\mathrm{dt}} \times 60\times \frac{\mathrm{P}1}{\mathrm{P}0} \frac{273}{(273+\mathrm{T})} \end{eqnarray*}

(2) \begin{eqnarray*}\mathrm{CCE}= \frac{\sum ({\mathrm{E}}_{\mathrm{i}}+{\mathrm{E}}_{\mathrm{i}+1})}{2} \times ({\mathrm{t}}_{\mathrm{i}+1}-{\mathrm{t}}_{\mathrm{i}})\times 24\end{eqnarray*}

where E is the CO_2_ flux of the soil surface (µg m^−2^ h^−1^); A is the area of the static chamber (76.0 cm^2^); V is the volume of static chamber (77.0 cm^3^); *ρ* is the standard atmospheric CO_2_ density (1.963 mg m^−3^); dc/dt is the CO_2_ concentration variation (10^−6^ min^−1^); P_0_ is the atmospheric pressure which is equal to the atmospheric pressure within the station under standard atmospheric conditions (1.013 × 10^5^Pa); P_1_ is static chamber’s the atmospheric pressure; T is the air temperature (°C); CCE is the cumulative CO_2_-C emissions (kg CO_2_ ha^−1^); t is the number of days after planting, and i is the number of the sample.

### Evapotranspiration

Evapotranspiration was computed following the method Ren et al., 2018a; Ren et al., 2018b),

(3) \begin{eqnarray*}\mathrm{ET}=P+I-\Delta S-S-SR\end{eqnarray*}

where ET is the evapotranspiration volume (mm); P is the effective precipitation in the experimental year (mm), provided by a meteorological station 10.0 m away from the study site; I is the irrigation volume (mm); ΔS is the change in soil water storage, calculated by the difference between the initial content of soil moisture and the latest value, which was measured every 7 days from planting date to harvest date; S is the seepage under the crop root zone (mm), which was negligible because the measured soil water value indicated that drainage was low; SR is the surface runoff which was negligible, because of a lack of heavy precipitation during the study period, and the presence of a 20 cm barrier above the soil surface which was built around each experimental plot to prevent surface runoff.

A neutron moisture meter (CNC 503D, Super Energy. Nuclear Technology Ltd., Beijing, China) was used to measure the volumetric moisture content. The measurement was taken every 10 cm from the soil surface to a depth of 120 cm. To reduce error, we calibrated the water content used an oven dry method of the topsoil (20 cm). The soil was dried at 105 °C in an oven until a constant weight was reached and compared to determine the soil moisture. The groundwater depth was greater than 5.0 m, so the influence of groundwater on water consumption was not considered in this study.

### Grain yield and yield compositions

In each plot, two 1.5 m sections of rows were randomly selected with three independent replicates to measure grain yield, spike numbers, and 1000-kernel weight at maturity. The kernel numbers per spike were measured using an additional 20 spikes. The winter wheat was harvested by hand.

### Yield carbon utilization efficiency

The YCUE was computed following the method (Liu et al., 2014),

(4) \begin{eqnarray*}\mathrm{Y CUE}= \frac{\mathrm{Y }}{\mathrm{CCE}} \end{eqnarray*}

where Y is the grain yield (g m^−2^) and CCE is the cumulative CO_2_-C emissions (g m^−2^).

### Water use efficiency

The WUE (kg/m^3^) was computed following the method (Li et al., 2015),

(5) \begin{eqnarray*}\mathrm{WUE}= \frac{\mathrm{Y }}{\mathrm{ET}} \end{eqnarray*}

where Y is grain yield of winter wheat (g/m^2^), and ET is the evapotranspiration during the experimental year (mm).

### Carbon emissions per unit water consumption

The WUE_CE_ (g m^−3^) was computed following the method (Hu et al., 2017),

(6) \begin{eqnarray*}\mathrm{WU}{\mathrm{E}}_{\mathrm{CE}}= \frac{\mathrm{CCE}}{\mathrm{ET}} \times 1000\end{eqnarray*}

where CCE is cumulative CO_2_-C emissions (g m^−2^), and ET is evapotranspiration (mm).

### Statistical analysis

Differences among treatments were assessed using analysis of variance (ANOVA) with a significance level of *α* = 0.05. When significance was observed, the least significant difference (LSD) post-hoc test was used to conduct multiple comparisons. The normality of variances was tested before performing the ANOVA. Microsoft Excel 2010 and SPSS were used to organize and analyze data, respectively.

## Results

### Cumulative CO_2_-C emissions

**Figure 3:**
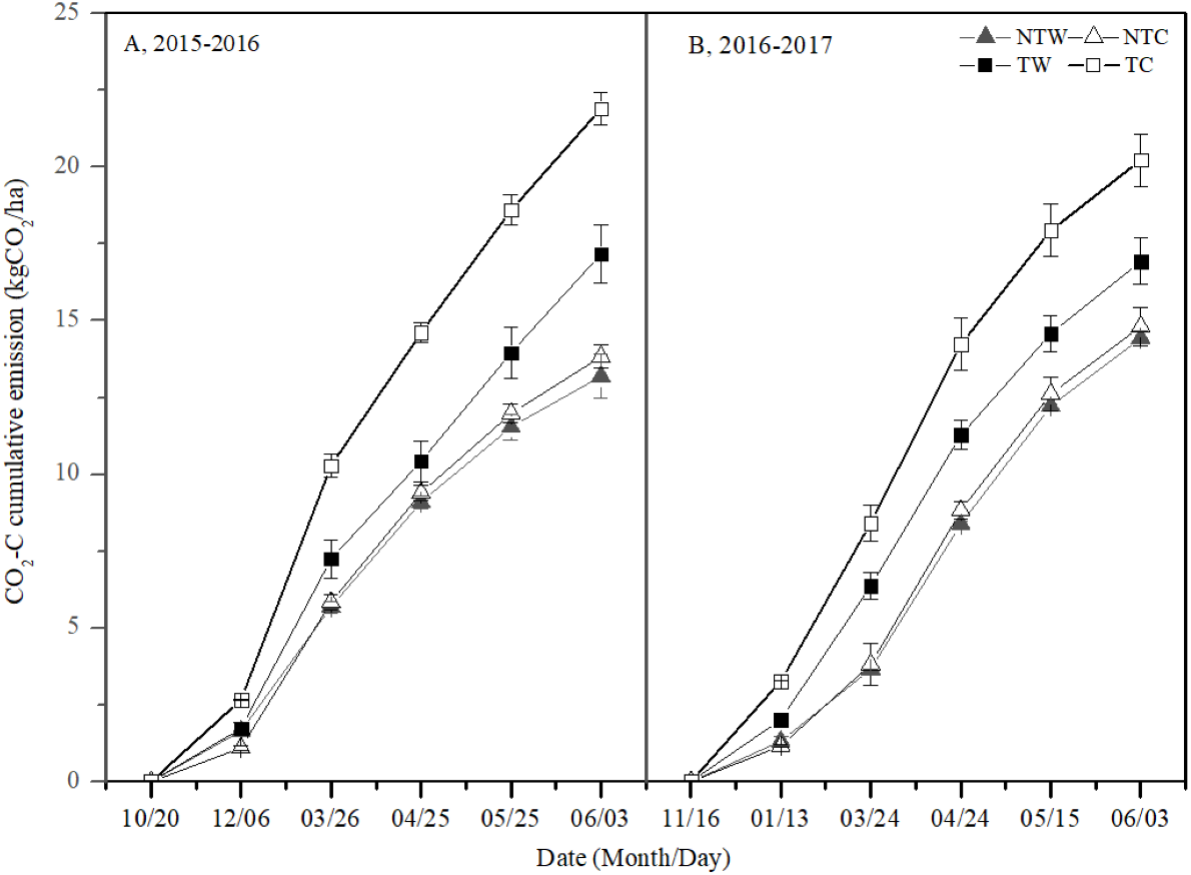
CO_2_-C cumulative emission in 2015–2016 and 2016–2017 winter wheat growing season. NTW, NTC, TW and TC represent no tillage with wide-precision planting, no tillage with conventional planting, conventional tillage with wide-precision planting, and conventional tillage with conventional planting. Vertical bars are standard errors, the maximum standard errors in 2015–2016 and 2016–2017 winter wheat growing seasons were 0.94 and 1.62, respectively.

The patterns of CCE were similar over the two growing seasons (Fig. 3). The TC treatment had the highest CCE, followed by the TW treatment and the NTC treatment. The NTW treatment had the lowest CCE. The CCE were lower in NT than in T (30.8 and 21.3%, respectively) and were lower in W than in C (15.0 and 10.5%, respectively) in the first and second growing seasons. Moreover, CCE were slightly lower in NTW than in NTC (4.6 and 2.6%, respectively) and were lower in TW than in TC (21.6 and 16.3%, respectively). In 2015**–**2016 and 2016**–**2017, CCE were lower in NTW than in TW (23.2 and 14.8%, respectively) and were lower in NTC than in TC (36.8 and 26.8%, respectively). There was a significant interaction between tillage methods and planting patterns. The NT treatment combined with the W treatment appeared to inhibit CO_2_-C emissions.

### Evapotranspiration

**Figure 4:**
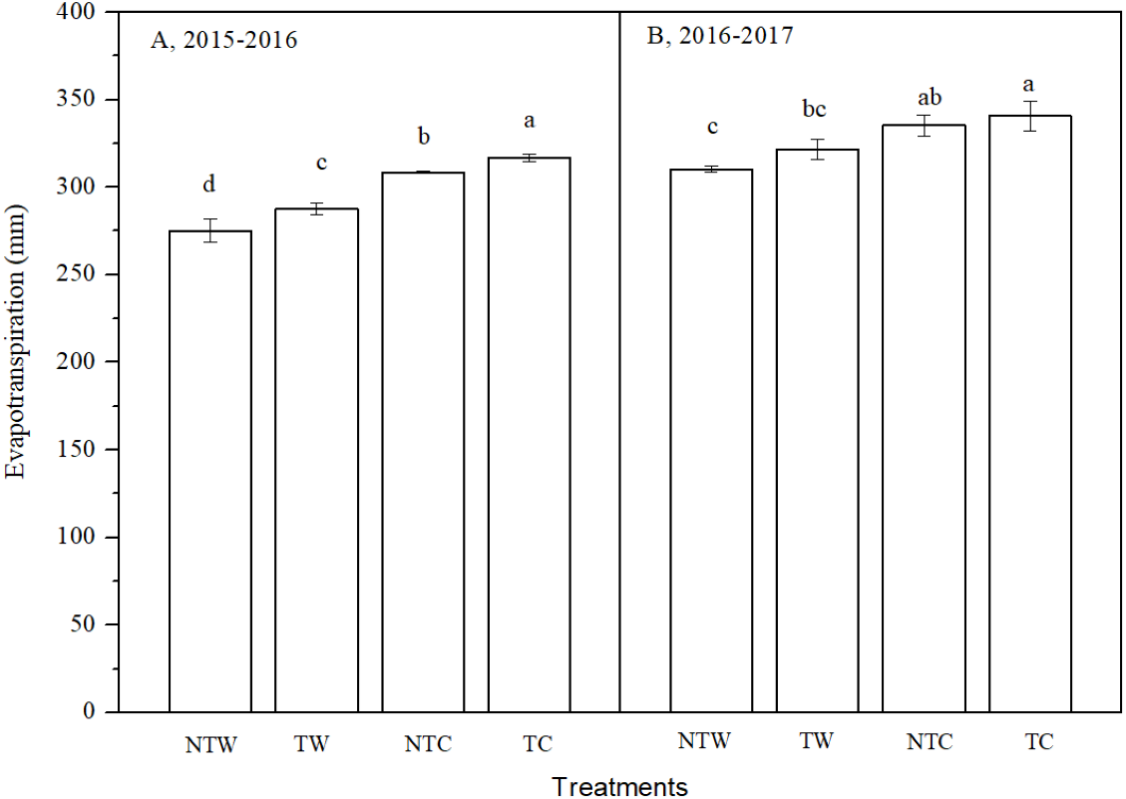
Evapotranspiration in 2015–2016 and 2016–2017 winter wheat growing season. NTW, NTC, TW and TC represent no tillage with wide-precision planting, no tillage with conventional planting, conventional tillage with wide-precision planting, and conventional tillage with conventional planting. Vertical bars are standard errors. Bars labeled at the top of the column with different letters are significantly (*P* < 0.05) different among treatments using LSD post–hoc test.

Evapotranspiration was similar in the two growing seasons (Fig. 4). The NTW treatment had the lowest evapotranspiration, followed by the TW treatment, and finally the TC treatment. Evapotranspiration in winter wheat ranged from 275.7 to 316.7 mm and 310.4 to 340.7 mm in 2015–2016 and 2016**–**2017, respectively. Evapotranspiration was slightly lower in NT than in T (3.4 and 2.5%, respectively) and it was lower in W than in C (10.0 and 6.5%, respectively). Furthermore, evapotranspiration was lower in NTW than in TW (4.4 and 3.5%, respectively), and it was lower in NTC than in TC (2.6 and 1.6%, respectively). NT and W appeared to decrease evapotranspiration in the two experimental years.

### Grain yield and yield components

Tillage pattern had a significant effect on winter wheat yield compositions (Table 1) in the two experimental years. Both the 1000-kernel weight and kernel numbers per spike were higher in the NT treatments than the T treatments (4.8 and 1.4%, and 5.7 and 7.1%, respectively, in 2015–2016 and 2016–2017). However, compared with T, NT had significantly lower spike numbers (12.0 and 21.7%, respectively). This meant that, overall, NT had a significantly lower winter wheat grain yield than T (9.0 and 9.5%, respectively).

**Table 1:** Yield and yield components in 2015–2016 and 2016–2017 winter wheat growing season.

Treatments	Spike numbers	Kernel numbers	1000-kernel	Grain
	(spikes m^−2^)	per spike	weight	yield
		(kernels spike^−1^)	(g)	(g m^−2^)
2015–2016				
Tillage				
NT	595.67b	42.68b	38.65a	655.13b
T	676.80a	40.39a	36.88b	719.57a
Plant pattern				
W	655.52a	40.72b	36.52b	700.26a
C	616.94b	42.35a	39.00a	674.45b
Coupling				
NTW	625.78b	42.02ab	37.78b	673.04ab
TW	685.26a	39.43c	35.26c	727.48a
NTC	554.17c	43.35a	39.35a	637.22c
TC	668.33a	41.35b	38.49b	711.67a
2016–2017				
Tillage				
NT	765.20b	42.61a	37.39a	738.84b
T	977.58a	39.80b	36.87a	816.22a
Plant pattern				
W	887.44a	40.37b	36.94a	806.73a
C	855.35b	42.04a	37.32a	748.33b
Coupling				
NTW	789.15b	41.49b	37.22ab	749.35b
TW	985.72a	39.25c	36.66b	864.11a
NTC	741.25c	42.73a	37.57a	728.33c
TC	969.44a	40.35bc	37.07ab	768.33b
Interaction				
Tillage × Genotypes	0.002	0.001	0.069	0.000

**Notes.**

NTC no tillage with conventional planting NTW no tillage with wide-precision planting TC conventional tillage with conventional planting TW conventional tillage with wide-precision planting

In each experiment year, different letters indicate significant differences tested by LSD posthoc test (*P* < 0.05).

The effect of planting pattern on yield compositions was significant (Table 1). Compared with C, the W treatments had fewer kernel numbers per spike (3.8 and 4.0% less, respectively) and lower 1000-kernel weights (6.4 and 1.0%, respectively), but it had more spikes (6.3 and 3.8%, respectively) and higher grain yields (by 3.8 and 7.8%, respectively).

The TW treatment had the highest grain yields (727.5 and 864.1 g m^−2^, respectively) and spike numbers (685.3 and 985.7 spike m^−2^, respectively), and had the lowest kernel numbers per spike (39.4 and 39.3 kernels spike^−1^, respectively) and 1000-kernel weights (35.3 and 36.7 g, respectively). Compared with that of TW, grain yields in TC, NTW, and NTC were significantly lower (2.2, 7.5, and 12.4%, respectively, in 2015**–**2016, and 11.1, 13.3, and 15.7%, respectively, in 2016**–**2017). It appears that the NT treatments had significant negative effects on grain yield and spike numbers and significant positive effects on kernel numbers per spike and 1000-kernel weight. The W treatments compensated for the NT treatments with regards to grain yield loss. The W treatments enhanced grain yields mainly through the increase of spike numbers.

### Yield carbon utilization efficiency

The YCUE was influenced by tillage methods and planting patterns in winter wheat, and ranged from 0.33 to 0.51 in 2015**–**2016 and from 0.38 to 0.52 in 2016**–**2017 (Table 2). NT had a significantly higher YCUE than that of T (32.4 and 13.3% in 2015**–**2016 and 2016**–**2017, respectively). Compared with C, W had a higher YCUE by 20.5 and 16.0% in the two experimental years, respectively. The YCUE in NTW, NTC, and TW was significantly higher than in TC by 54.5, 39.4, and 27.2%, respectively, in 2015**–**2016 and by 36.8, 28.9, and 34.2%, respectively, in 2016**–**2017.

**Table 2:** Water use efficiency and yield carbon utilization efficiency in the 2015–2016 and 2016–2017 winter wheat growing season.

Treatments	Yield carbon utilization efficiency	Water use efficiency (kg m^−3^)	Carbon emissions per unit water consumption (g m^−3^)	
2015–2016			
Tillage			
NT	0.49a	2.26b	46.39b	
T	0.37b	2.39a	64.41a	
Plant pattern			
W	0.47a	2.49a	53.83b	
C	0.39b	2.16b	56.98a	
Coupling			
NTW	0.51a	2.45b	47.98c	
TW	0.42c	2.54a	59.68b	
NTC	0.46b	2.06d	44.81d	
TC	0.33d	2.25c	69.14a	
2016–2017			
Tillage			
NT	0.51a	2.29b	45.34b	
T	0.45b	2.47a	56.02a	
Plant pattern			
W	0.51a	2.55a	49.58b	
C	0.44b	2.21b	51.78a	
Coupling			
NTW	0.52a	2.41b	46.50c	
TW	0.51ab	2.69a	52.65b	
NTC	0.49b	2.17d	44.18d	
TC	0.38c	2.26c	59.38a	
Interaction			
Tillage × Genotypes	0.001	0.087	0.000	

**Notes.**

NTC no tillage with conventional planting NTW no tillage with wide-precision planting TC conventional tillage with conventional planting TW conventional tillage with wide-precision planting

In each experiment year, different letters indicate significant differences tested by LSD posthoc test ( *P* < 0.05).

### Water use efficiency

The NT treatments had lower WUE than the T treatments (5.4 and 7.3% in 2015–2016 and 2016–2017, respectively), and W had higher WUE than in C (15.3 and 15.4% in 2015–2016 and 2016–2017, respectively) (Table 2). In the two experimental years, NTW had higher WUE than in NTC (18.9 and 11.1%, respectively), and TW had higher WUE than in TC (12.9 and 19.0%, respectively). It appeared that the NT treatments decreased WUE, but the W treatments had a compensatory effect on the NT treatments with respect to WUE.

### Carbon emissions per unit water consumption

In the two experimental years, WUE_CE_ was significantly lower in NT than in T (by 28.0 and 19.1%, respectively), and WUE_CE_ was significantly lower in W than in C (5.5 and 4.2% in 2015–2016 and 2016–2017, respectively) (Table 2). WUE_CE_ was significantly lower in NTW than in that of TW (19.6 and 11.7%, respectively) and significantly lower in NTC than in that of TC (35.2% and 25.6%, respectively). These results suggest that both the NT and W treatments reduced WUE_CE_.

## Discussion

### CO_2_ emissions

In this study, NTW had lowest CCE because of the beneficial interaction of tillage methods and planting patterns, and TC had highest CCE. For tillage methods, tilling intensively disturbed the soil, broke down soil aggregates, and exposed organic matter that was protected by soil aggregates for microbial decomposition (Six, Elliott & Paustian, 2000); the root length density was significantly higher in T than NT treatments deeper than 10 cm in the soil profile (Qin, Stamp & Richner, 2006). These factors all increased carbon emissions. During our two-year experimental period, CCE was higher in T than NT by 1.3 to 1.5 times. This finding was consistent with a previous study, which indicated that total seasonal CO_2_ emissions were 1.6 times higher in T soils than NT soils (Zhang et al., 2016). Compared with C, W increased the photosynthetically active radiation capture ratio at 40 and 60 cm above the ground, and improved the leaf area index (Zhang et al., 2013). This then decreased the exposure and temperature of the soil surface (Liang & Richards, 2012), and reduced soil respiration. The reduction in CCE from combining planting patterns with tillage methods will improve the methods used for mitigating CO_2_ emissions from agricultural soil.

Differences were found in CCE under T between the two years, which may be related to precipitation. In 2015–2016, total precipitation from April to harvest was 106.3 mm (67.84% of the whole growing season), while in 2016–2017 it was 70.3 mm (44.69%) in 2016–2017. A loose soil structure and suitable water content enhanced microbial activity and root system activity, resulting in higher soil respiration (Zhang et al., 2011). Hence, leading to higher CCE under T in 2015–2016 than 2016–2017.

NT reduced carbon emissions, thereby increasing soil organic carbon storage (Huang et al., 2015; Liu et al., 2015). W also reduced CCE, but it was unclear whether W increased soil carbon. More research is needed into the relationship between soil carbon emissions, soil carbon storage under W.

### Grain yield and yield carbon utilization efficiency

The responses of different ecosystems to agronomic management practices vary (Brouder & Gomez-Macpherson, 2014). In general, the reasons for crop yield losses under NT are plant diseases (Wang et al., 2020), lower flag leaf fluorescence parameters and leaf area index (Liu et al., 2019), and reduction of spike numbers (Ren et al., 2018b). Winter wheat spike numbers were lower in NT than in T in this study. However, crop yield also varied under different planting patterns. Spike numbers in W treatments were significantly higher than C treatments (Li et al., 2015), which was consistent with the finding of our study, and this was the crucial factor which affected grain yield (Zhao et al., 2013). Although, NT decreased spike numbers, W enhanced spike numbers. Compared with TC, NTC decreased spike numbers by 17.1% while NTW decreased spike numbers by only 6.4% in the 2015–2016 growing season. The difference in spike numbers between NTC and TC, as well as NTW and TC, provided support for the compensatory effect of W under NT on the reduction of grain yield.

Spike numbers were higher in the second experimental year than in the first. The total precipitation was same from Nov. to Feb. in the two experimental years, but precipitation frequency was different. There was no precipitation in Dec. and Jan. in 2015–2016; however, precipitation occurred in all months in 2016–2017. The months from Nov. to Feb. are vital for tiller formation on winter wheat. A certain amount of in-season soil moisture is necessary to meet the water demands of crop components and enable root development to access the deeper soil moisture, and the well-distributed growing season precipitation is important for achieving higher grain yield (Thapa et al., 2020). Grain yield was closely related with tiller numbers. Precipitation frequency may explain the difference in spike numbers in the two experimental years.

Grain yield and CCE had an effect on YCUE. Although NT decreased grain yield, it also decreased CCE. T improved the porosity of the soil surface and the activity of root; this combined with the wet conditions of the soil, increases carbon emissions, thereby leading to a decrease in YCUE. W increased grain yield and decreased CCE. Hence, the NTW treatment had the highest YCUE among the other treatments. The interaction between tillage methods and planting patterns was significant for YCUE.

Although the yields of winter wheat in the NT other treatments were lower than those of the T treatments in the NCP, W had a compensatory effect on grain yield and increased YCUE. To mitigate GHG emissions, NT and W together appeared to provide a viable approach for cleaner production.

### Water use

NT decreased evapotranspiration in this study, which was similar to Huang et al. (2012), because NT increased interception of precipitation, and it reduced water absorption because of the lower root volume (Ali et al., 2018). WUE was lower in NT than T, mostly because of the reduction in grain yield under NT, which was similar to previous study (Ren et al., 2018a; Ren et al., 2018b). W had higher WUE than C, mostly because of higher improving grain yield, as well as, reduced evapotranspiration. The reduction of evapotranspiration under W may be related to the change of sowing width (6–8 cm under W; 3–5 cm under C), which could increase the leaf area index (Zhao et al., 2013), reduce the exposure of the soil surface, and decrease evaporation between crop rows. The W treatments appeared to have a compensatory effect on WUE under NT treatments, and the combination may provide a more suitable strategy for sustainable agriculture.

In Northwestern China, another study found that reduced tillage in wheat production had mean WUE_CE_ values of 2.3 kg C ha^−1^mm^−1^, which was 4.7% lower than that of T (Hu et al., 2015), much less than the 28.0 and 19.0% differences observed for the 2015–2016 and 2016–2017 seasons, respectively, in the present study. Differences in temperature and precipitation between the study areas probably caused this difference. In the previous study, spring wheat was planted in Mar. and harvested in Jul. in Northwestern China and in the present study, winter wheat was planted in Oct. in the first year and harvested in Jun. in the second year in the NCP. There were large differences in the temperature and precipitation conditions experienced by the crops.

### Limitations and future research

Because of climate variability and soil heterogeneity, there are high temporal and spatial differences in GHG emissions. We only measured the CO_2_-C of the soil surface in this study, so we could not better explain trends in total GHG emissions. Decreasing carbon emissions might also be related to the soil organic carbon pool and root growth in the W treatment. Further investigations into crop root development and the soil carbon sequestrations under different tillage and planting methods should be considered in the NCP area. However, we have shown that there is a clear difference in carbon emissions and WUE under different planting patterns and tillage methods. This provides options for investigating cleaner agricultural production. In the long run, the combination of NT and W may benefit food security and environment conditions. This study was an analogous tillage experiment, it was not a mechanical tillage field experiment. We recommend that a field experiment should be established to verify the results of our research.

## Conclusion

NT reduced carbon emissions and water consumption, but also reduced the grain yield of winter wheat. W decreased soil carbon emissions in NT and led to higher grain yields and WUE than C, mainly because of the increase of spike numbers. Therefore, the W treatment appeared to have a compensatory effect on grain yield and WUE under NT. NT and W can significantly reduce WUE_CE_. Thus, in the NCP, the combination of NT and W can reduce soil carbon emissions and increase the efficient use of water. Our finding provides theoretical support for more sustainable production of winter wheat crops.

##  Supplemental Information

10.7717/peerj.9912/supp-1 Supplemental Information 1 CO_2_-C cumulative emission in 2015–2016 and 2016–2017 winter wheat growing season NTC, no tillage with conventional planting; NTW, no tillage with wide-precision planting; TC conventional tillage with conventional planting; TW conventional tillage with wide-precision planting.

10.7717/peerj.9912/supp-2 Supplemental Information 2 Yield and yield components and water use efficiency in 2015–2016 and 2016–2017 winter wheat growing season TW, NTC, TW and TC represent no tillage with wide-precision planting, no tillage with conventional planting, conventional tillage with wide-precision planting, and conventional tillage with conventional planting.

10.7717/peerj.9912/supp-3 Supplemental Information 3 Daily precipitation and mean air temperature in the 2015–2016 and 2016–2017 winter wheat growing seasons, at the Agricultural Experiment Station of Shandong Agricultural University Total precipitation in Oct was from seeding time to end of Oct and total precipitation in Jun was from beginning of Jun to the harvest time.

## Additional Information and Declarations

### Data Availability

The following information was supplied regarding data availability: The raw measurements are available in the Supplemental Files.
